# Population genomics of local adaptation versus speciation in coral reef fishes (*Hypoplectrus* spp, Serranidae)

**DOI:** 10.1002/ece3.2028

**Published:** 2016-02-26

**Authors:** Sophie Picq, W. Owen McMillan, Oscar Puebla

**Affiliations:** ^1^ Evolutionary Ecology of Marine FishesGEOMAR Helmholtz Centre for Ocean Research KielDüsternbrooker Weg 2024105KielGermany; ^2^ Faculty of Mathematics and Natural SciencesUniversity of KielChristian‐Albrechts‐Platz 424118KielGermany; ^3^ Smithsonian Tropical Research InstituteApartado Postal 0843‐03092PanamáRepública de Panamá

**Keywords:** Fish, local adaptation, marine, RAD sequencing, speciation

## Abstract

Are the population genomic patterns underlying local adaptation and the early stages of speciation similar? Addressing this question requires a system in which (i) local adaptation and the early stages of speciation can be clearly identified and distinguished, (ii) the amount of genetic divergence driven by the two processes is similar, and (iii) comparisons can be repeated both taxonomically (for local adaptation) and geographically (for speciation). Here, we report just such a situation in the hamlets (*Hypoplectrus* spp), brightly colored reef fishes from the wider Caribbean. Close to 100,000 SNPs genotyped in 126 individuals from three sympatric species sampled in three repeated populations provide genome‐wide levels of divergence that are comparable among allopatric populations (*F*_st_ estimate = 0.0042) and sympatric species (*F*_st_ estimate = 0.0038). Population genetic, clustering, and phylogenetic analyses reveal very similar patterns for local adaptation and speciation, with a large fraction of the genome undifferentiated (*F*_st_ estimate ≈ 0), a very small proportion of *F*_st_ outlier loci (0.05–0.07%), and remarkably few repeated outliers (1–3). Nevertheless, different loci appear to be involved in the two processes in *Hypoplectrus*, with only 7% of the most differentiated SNPs and outliers shared between populations and species comparisons. In particular, a tropomyosin (*Tpm4*) and a previously identified hox (*HoxCa*) locus emerge as candidate loci (repeated outliers) for local adaptation and speciation, respectively. We conclude that marine populations may be locally adapted notwithstanding shallow levels of genetic divergence, and that from a population genomic perspective, this process does not appear to differ fundamentally from the early stages of speciation.

## Introduction

Whether populations are adapted to local conditions and, if so, through what mechanisms are fundamental questions in evolutionary ecology (Williams 1966; Kawecki and Ebert 2004; Savolainen et al. 2013). This is particularly true in the marine environment, where absolute barriers to the movement of organisms are few and planktonic larval stages provide potential for extensive dispersal. Are marine populations able to adapt to local environmental conditions in such a potentially high gene‐flow context? This is not only a basic question but also an applied one, as the occurrence of locally adapted marine populations has far‐reaching implications for management, conservation, and the ability to cope with global change (Conover et al. 2006; Hauser and Carvalho 2008; Munday et al. 2013).

Common gardens and reciprocal transplants can provide direct evidence of local adaptation. These approaches suggest that local adaptation is not uncommon in marine species, even in the presence of planktonic dispersal, and sometimes at small spatial scales (Sotka 2005; Sanford and Kelly 2011). Nevertheless, such experiments can be challenging to implement in highly mobile or hard‐to‐breed species, which are both common in the marine environment. In addition, the selective factors involved are not always identified and the specific traits underlying local adaptation as well as their genomic bases are almost universally unknown.

Genome scans provide the opportunity to identify the genetic footprints of local adaptation in natural populations, even in the absence of a priori hypotheses about the selective factors and specific traits involved (Savolainen et al. 2013; Tiffin and Ross‐Ibarra 2015). Such studies are starting to accumulate in marine fishes (Lamichhaney et al. 2012; Milano et al. 2014), with the Atlantic cod leading the pack (Bradbury et al. 2013; Hemmer‐Hansen et al. 2013; Berg et al. 2015). Although a number of factors unrelated to adaptation can generate false positives in genome scan data (Pérez‐Figueroa et al. 2010; Bierne et al. 2011, 2013; Vilas et al. 2012; Lotterhos and Whitlock 2014), all genome scan studies on marine fishes identify candidate loci for local adaptation, with temperature and salinity emerging as usual suspects regarding the selective factors involved.

An important aspect of local adaptation is its potential to initiate, facilitate, or drive speciation (Gavrilets 2003; Kawecki and Ebert 2004; Nosil 2012; Savolainen et al. 2013; Tiffin and Ross‐Ibarra 2015), and the ecological hypothesis of speciation (Schluter 2001) specifically postulates that speciation may result as a by‐product of local adaptation. Nevertheless, marine local adaptation and speciation are often considered in isolation of each other. Here, we aim to bridge this gap by asking whether the population genomic patterns underlying local adaptation and speciation are comparable. Addressing this question requires a system in which (i) local adaptation and the early stages of speciation can be clearly identified and distinguished, (ii) the amount of genetic divergence driven by the two processes is similar (thereby eliminating the confounding factor posed by divergence when species are more diverged than populations), and (iii) comparisons can be repeated both taxonomically (for local adaptation) and geographically (for speciation).

The hamlets (*Hypoplectrus* spp, Serranidae) constitute just such a system. These reef fishes from the wider Caribbean are known for their striking variation in color pattern (Thresher 1978; Fischer 1980; Domeier 1994; Lobel 2011). Seventeen species have been described to date, which differ essentially in terms of color pattern. A combination of natural selection on color pattern (Thresher 1978; Puebla et al. 2007) and sexual selection (Puebla et al. 2012a) has been put forward to explain the origin and maintenance of species within the radiation. The hamlets are highly sympatric, with up to nine species found on a single reef. The different hamlet species spawn at the same time and in the same areas, often within sight of each other. Nevertheless, spawning is strongly assortative with respect to color pattern, with >98% of spawnings occurring among members of the same species (Fischer 1980; Barreto and McCartney 2007; Puebla et al. 2007, 2012a). Hamlets from the Gulf of Mexico appear to be well diverged (Victor 2012; Tavera and Acero 2013), but species within the Caribbean are extremely similar from a genomic perspective, with *F*_st_ estimates between sympatric species ranging between zero and 0.080 at microsatellite loci (McCartney et al. 2003; Puebla et al. 2007, 2012a). RAD analysis of three sympatric species repeated in three Caribbean populations confirmed the microsatellite results and identified a very small proportion of SNPs (0.05%) as *F*_st_ outliers between sympatric species (Puebla et al. 2014). Remarkably, a single SNP was identified as an outlier in repeated populations for the same species pair (repeated outlier). A mini‐contig assembled de novo around this SNP mapped uniquely to the genomic region between the *HoxC10a* and *HoxC11a* genes in 10 teleost species, suggesting a possible role for *Hox* gene evolution in hamlet speciation.

Caribbean hamlets also present low level of genetic structure within species, with *F*_st_ estimates among allopatric populations ranging between 0.006 and 0.047 at microsatellite loci (McCartney et al. 2003; Puebla et al. 2008, 2009). Such low levels of genetic structure are typical of marine species and raise the question as to whether populations are able to adapt to local conditions. Differences in morphology (Thresher 1978; Aguilar‐Perera 2004), diet (Whiteman et al. 2007b; Holt et al. 2008), and behavior (O. Puebla, pers. obs.) have been reported between Caribbean hamlet populations, but it is unclear whether these differences are plastic or adaptive, and if they are adaptive, what selective factors might drive them.

Here, we reanalyze the RAD data presented in Puebla et al. (2014), but comparing allopatric populations instead of sympatric species. We hypothesize that if hamlets are locally adapted, outlier loci should occur among populations, and consistently so in the three species (repeated outliers). In addition, if such repeated outliers are present and can be mapped to known genomic regions, their identity may give us a hint as to what selective factors may be important for local adaptation. Finally, we contrast the population genomic patterns underlying local adaptation to the population genomic patterns underlying speciation described in Puebla et al. (2014). Given the distinct natural histories (and hence selective factors) underlying the two processes, we hypothesize that the loci associated with local adaptation should differ from the loci associated with speciation.

## Materials and Methods

This study is based on the same dataset presented in Puebla et al. (2014), but comparing allopatric populations instead of sympatric species. In order to allow direct comparisons between local adaptation and speciation, the same methodology used in Puebla et al. (2014) is followed here. An overview of the methods is provided below and we refer to Puebla et al. (2014) for details. New simulations and new analyses of linkage disequilibrium are described in detail.

### Sampling and genotyping

This study is based on nine samples including three sympatric species (the barred hamlet *Hypoplectrus puella*, the black hamlet *Hypoplectrus nigricans*, and the butter hamlet *Hypoplectrus unicolor*) from three locations (Belize, Honduras, and Panama), with 14 individuals per sample (total 126 individuals). This sampling design provides the opportunity to explore the population genomic patterns of local adaptation (between allopatric populations within species) and speciation (between sympatric species) within a single system, and to repeat comparisons both taxonomically (in three species for local adaptation) and geographically (in three populations for speciation).

Libraries were prepared following the restriction site‐associated DNA (RAD) sequencing protocol by Etter et al. (2011) and sequenced as detailed in Puebla et al. (2014). In order to compare the results provided by RAD sequencing and microsatellites, microsatellite data from Puebla et al. (2007, 2012a) were reanalyzed for the populations and species considered in this study (10 loci, 50 individuals per sample).

### Raw sequences filtering and assembly

Filtering of the raw sequences included the removal of low‐quality reads, reads with an ambiguous index or *Sbf*I restriction site, and reads including adapter sequence as detailed in Puebla et al. (2014). Pairs of paired‐end reads that matched exactly were filtered out, as these are expected to represent PCR clones in the vast majority of cases.

Reads were assembled de novo using Stacks (Catchen et al. 2011, 2013). The number of raw reads required to form a stack (*m*) was set to three and the number of allowed nucleotides mismatch between two stacks (*M*) to two, which is in line with the guidelines provided by Catchen et al. (2013), Ilut et al. (2014), and Mastretta‐Yanes et al. (2015). In order to test the robustness of the results to these assembly parameters, the main analyses were rerun with *m *=* *3 *M *=* *3, *m *=* *4 *M *=* *2, *m *=* *5 *M *=* *4, and *m *=* *10 *M *=* *4.

### Population genetic statistics

In order to allow direct comparisons with previous results on speciation, the same moderate filtering used in Puebla et al. (2014) was applied to the dataset unless stated otherwise for specific analyses.

Analyses were also repeated with more stringent filtering and, as indicated throughout the Results, similar genomic patterns were obtained.

Samples were either pooled by location (Belize, Honduras, Panama, *n *=* *42 individuals per location), retaining stacks with coverage ≥10x in ≥15 individuals per location in ≥2 locations, or considered individually (*n *=* *14 individuals per sample), retaining stacks with coverage ≥10x in ≥5 individuals per group in ≥7 samples. *F*_st_ were estimated following a standard analyses of variance (ANOVA) approach (Weir and Cockerham 1984) using Genepop version 4.2.1 (Rousset 2008).

### Clustering analyses

Clustering analyses (Pritchard et al. 2000) were performed to further explore genetic structure. The same filtering as above was used, but this time considering a single SNP per stack (the first one). The admixture model with correlated frequencies was considered (Falush et al. 2003), and species/location information was not used to preassign individuals to clusters or to improve clustering. *K* was set from one to 10 and 10 replicate analyses (100,000 MCMC burning steps followed by 100,000 iterations) were run for each value of *K*. Structure Harvester (Earl and vonHoldt 2012) was used to summarize the results from the 100 runs performed for each analysis. Both ln Pr(*X*|*K*) and the ad hoc statistic Δ*K* (Evanno et al. 2005) were used to infer the number of clusters present in the dataset.

**Figure 1:**
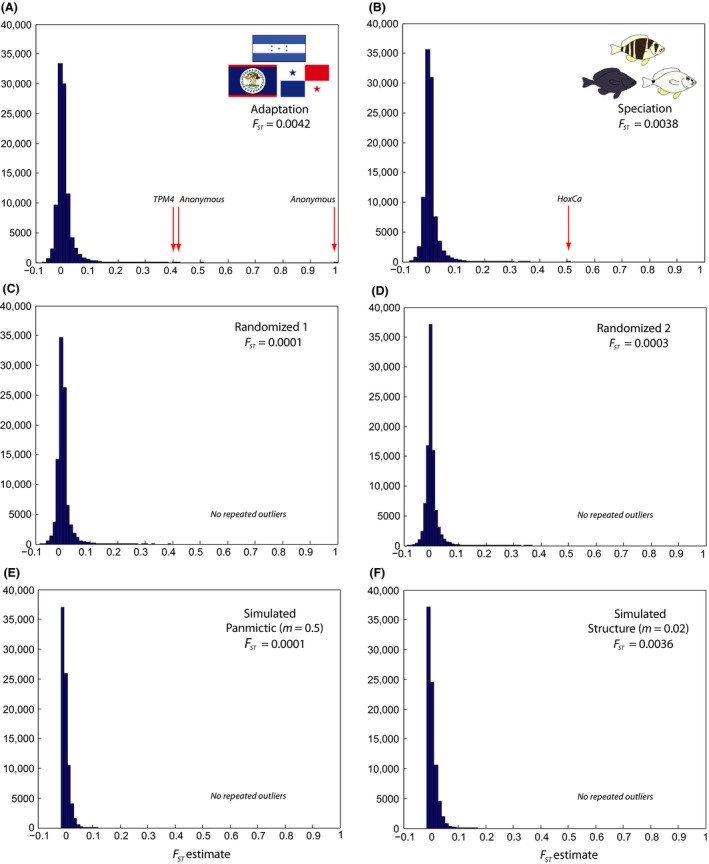
Frequency distribution of individual SNP *F*_st_ estimates (A) among locations (Belize, Honduras, and Panama, 97,962 SNPs), (B) among species (*Hypoplectrus puella*,* H. nigricans*, and *H. unicolor*, 96,418 SNPs, from Puebla et al. 2014), (C, D) among random groups (95,274 and 95,309 SNPs, respectively), and (E, F) for simulated data (panmictic, migration rate *m *=* *0.5, and structure, migration rate *m *=* *0.02, 80,000 loci in both cases). Repeated outliers highlighted with red arrows.

Genetic structure was further analyzed with different SNP subsets. These were established according to global *F*_st_ estimates among locations (Fig. 1A), considering the interval above the 90^th^ percentile (*F*_st_ ≥ 0.0266, 8038 SNPs), between the 80^th^ and 90^th^ percentiles (0.0127 ≤ *F*_st_ <0.0266, 8467 SNPs), between the 70^th^ and 80^th^ percentiles (0.0047 ≤ *F*_st_ < 0.0127, 8424 SNPs), between the 60^th^ and 70^th^ percentiles (−0.0006 ≤ *F*_st_ < 0.0047, 8034 SNPs), and below the 60^th^ percentile (*F*_st_ < −0.0006, 33,216 SNPs). This approach should be considered with caution, as there is some circularity in the process of selecting the most diverged SNPs to then explore genetic structure. Here, the most differentiated SNPs were selected to infer roughly how many and which SNPs were consistently differentiated among populations, and compare them with the number and identity of SNPs that were consistently differentiated among species (Puebla et al. 2014).

### SNP trees

In order to also adopt a phylogenetic perspective, SNPs were used to generate maximum‐likelihood trees. Preliminary analyses indicated that individuals with a high proportion of missing data contributed disproportionally to reduce bootstrap support values, so individuals with >20% missing data (mostly black and barred hamlets from Panama due to lower sequencing coverage in these populations) were filtered out. RAxML version 8.0.5 (Stamatakis 2014) was used for these analyses, implementing the GTR+G model with ascertainment bias correction and a rapid bootstrap procedure (Stamatakis et al. 2008) with 100 replicates per run. Analyses were run with the entire SNP dataset, and repeated with the same SNP subsets considered for the clustering analyses. Trees were generated with Dendroscope version 3.2.1 (Huson and Scornavacca 2012).

### Linkage disequilibrium network analysis

Linkage disequilibrium network analysis (LDna, Kemppainen et al. 2015) was performed to explore patterns of linkage disequilibrium (LD) in the dataset. Briefly, LDna starts from a matrix of pairwise LD estimates among loci and partitions loci into clusters, in which vertices represent loci, and edges LD values that are above a given threshold. The order in which clusters merge with decreasing LD threshold is represented as a tree where branches correspond to clusters, and nodes merging events. Change in median LD in a cluster at merging is measured by *λ*, and *λ* values exceeding the median by a user‐defined multiple *φ* of the median absolute deviation and containing at least |*E*|min edges (user‐defined also) identifies outlier clusters. Outlier clusters that do not have any other outlier clusters nested within them are defined as single‐outlier clusters (SOCs). We hypothesized that population structure should result in admixture LD when considering the entire dataset, and that clusters of loci in LD should differentiate populations and species.

Preliminary analyses indicated that LDna is sensitive to the occurrence of missing data, rare alleles (present in only one individual per sample), loci with heterozygosities >0.5, and that computation time for the calculation of the initial LD matrix becomes very long for >10,000 loci. LDna analyses were therefore restricted to black and barred hamlets from Honduras and Belize (which had highest sequencing coverage), filtering loci with coverage ≥20x in at least 11 individuals in all populations, removing loci with rare alleles and heterozygosities >0.5 and considering a single SNP per stack, which resulted in 10,734 SNPs. Global *F*_st_ among the four samples was estimated for each SOC identified, and a DAPC analysis with the four samples as groups was run for each SOC using Adegenet version 1.4‐2 (Jombart et al. 2010).

### *F*_st_ outlier analyses

Outlier scans were performed to identify SNPs that may be under selection. Bayescan version 2.1 (Foll and Gaggiotti 2008) was used for these analyses, with default parameters for run length and the prior odds for the neutral model set to 10 (default value) and 100. A locus was considered to be an outlier if it had a *q*‐value <0.2, corresponding to an expected false discovery rate of 20%.

Paired‐end reads were used to assemble mini‐contigs around the repeated outlier SNPs using Velvet version 1.2.03 (Zerbino and Birney 2008). Matches to the consensus sequences were searched using megablast on the NCBI server (http://www.ncbi.nlm.nih.gov/blast) and Blastn searches to the teleost genomes available on the Ensembl genome browser (Flicek et al. 2014, http://www.ensembl.org/index.html). Blast searches were also performed for the consensus sequence of all stacks that included nonrepeated outlier SNPs.

### Randomizations and simulations

In order to complement and better interpret our results, part of the analyses were repeated on randomized and simulated datasets. For the randomizations, the 126 samples were grouped into three random ‘species' from three random ‘locations' (nine samples total). Simulations were performed with SimuPOP version 1.1.4 (Peng and Kimmel 2005), considering an island model with nine populations of 1000 individuals each. Two scenarios were simulated, one with migration rate *m *=* *0.5 (‘panmictic') and one with *m *=* *0.02 (‘structure', which results in levels of genetic structure (*F*_st_ ≈ 0.004) that are similar to those observed in the real dataset). Each individual carried 80,000 diallelic unlinked loci with a mutation rate *μ* of 1E‐9. As for the real dataset, 14 individuals were sampled per population. Simulations were repeated three times and sampled 10 times each, resulting in a total of 30 datasets per scenario.

## Results

### Raw sequences filtering and assembly

A total of 565,253,125 reads of 101 bp each were retained after filtering, corresponding to 83.9% of the raw reads (see Puebla et al. 2014 for details). The main assembly (*m *=* *3 *M *=* *2) provided an average of 53,811 stacks per sample, with a mean coverage per stack of 31x before SNP filtering. The number of stacks decreased with increasing *m* and *M* parameter values, which is expected (Catchen et al. 2013). Nevertheless, similar global *F*_st_ estimates (0.0042–0.0044) and proportions of outliers (0.06–0.07%) were provided by the five assemblies with different combinations of *m* and *M* parameters (Table S1).

### Population genetic statistics

A total of 53,924 stacks were retained after pooling samples by location and filtering, providing 97,962 SNPs (i.e., 1.8 SNP per stack on average). Population genetic statistics are presented in Table S2. Considering all nucleotides, global diversity (*π*) and heterozygosity were estimated to 0.00240 and 0.00178, respectively, close to the values of 0.0036 and 0.00187 reported for sticklebacks (Hohenlohe et al. 2010). Global *F*_st_ among the three locations was estimated to 0.0042 when considering all SNPs. Close estimates of 0.0045, 0.0044, and 0.0039 were obtained when considering a single SNP per locus (the first one), removing loci with rare alleles (present in only one individual per location), or applying more stringent filtering (loci present in ≥32 individuals per location instead of 15), respectively. The distribution of SNP *F*_st_ estimates presented a sharp mode close to zero and a shallow tail extending to a value of one (Fig. 1A). *F*_st_ among the three locations was estimated to 0.0063 for *H. unicolor*, 0.0065 for *H. puella*, and 0.0131 for *H. nigricans*. Microsatellite data from the same populations provided close *F*_st_ estimates of 0.0034 for all species, 0.0032 for *H. puella*, and 0.0084 for *H. nigricans* (Table 1).

**Table 1:** *F*_st_ estimates among Belize, Honduras, and Panama in *Hypoplectrus puella*,* H. nigricans*, and *H. unicolor* at 10 microsatellite loci, 97,962 SNPs, and at the three repeated outliers identified in this study. *n* sample size, n/a data not available, – coverage below filtering criteria for these SNPs in these populations

Species	Location		*F*_st_ estimate (sample size)				
			10 *μ* satellite loci	97,962 SNPs	SNP 39,894 (*Tpm4*)	SNP 55,313 (anonymous)	SNP 38,220 (anonymous)
All species	All locations		0.0034 (*n *=* *418)	0.0042 (mean *n *=* *79.5)	0.3827 (*n *=* *92)	0.4146 (*n *=* *89)	1.0000 (*n *=* *43)
*H. puella*	All locations		0.0032 (*n *=* *154)	0.0065 (mean *n *=* *29.3)	0.3485 (*n *=* *33)	0.5178 (*n *=* *28)	1.0000 (*n *=* *20)
*H. nigricans*	All locations		0.0084 (*n *=* *156)	0.0131 (mean *n *=* *27.9)	0.4932 (*n *=* *26)	0.4162 (*n *=* *26)	1.0000 (*n *=* *14)
*H. unicolor*	All locations		n/a	0.0063 (mean *n *=* *26.1)	0.3875 (*n *=* *31)	0.5366 (*n *=* *30)	1.0000 (*n *=* *20)
*H. puella*	Belize	Honduras	0.0021 (*n *=* *100)	0.0050 (mean *n *=* *25.2)	0.4933 (*n *=* *27)	0.5178 (*n *=* *28)	1.0000 (*n *=* *15)
*H. nigricans*	Belize	Honduras	0.0059 (*n *=* *102)	0.0135 (mean *n *=* *24.3)	0.4933 (*n *=* *26)	0.4162 (*n *=* *26)	1.0000 (*n *=* *14)
*H. unicolor*	Belize	Honduras	n/a	0.0092 (mean *n *=* *15.2)	0.7108 (*n *=* *17)	0.8674 (*n *=* *18)	1.0000 (*n *=* *14)
*H. puella*	Honduras	Panama	0.0046 (*n *=* *104)	0.0222 (mean *n *=* *19.0)	0.1871 (*n *=* *20)	–	0.0000 (*n *=* *12)
*H. nigricans*	Honduras	Panama	0.0059 (*n *=* *105)	0.0361 (mean *n *=* *18.4)	–	–	–
*H. unicolor*	Honduras	Panama	0.0011 (*n *=* *108)	0.0090 (mean *n *=* *17.2)	0.1035 (*n *=* *21)	0.3970 (*n *=* *18)	0.0000 (*n *=* *13)
*H. puella*	Belize	Panama	0.0056 (*n *=* *104)	0.0213 (mean *n *=* *18.2)	0.0360 (*n *=* *19)	–	1.0000 (*n *=* *13)
*H. nigricans*	Belize	Panama	0.0132 (*n *=* *105)	0.0493 (mean *n *=* *17.1)	–	–	–
*H. unicolor*	Belize	Panama	n/a	0.0069 (mean *n *=* *20.1)	0.3545 (*n *=* *24)	0.2597 (*n *=* *24)	1.0000 (*n *=* *13)

When considering the nine samples independently, a total of 31,059 stacks were retained after filtering, providing 55,195 SNPs. *F*_st_ estimates among populations ranged between 0.0053 (*H. puella* Belize/Honduras) and 0.0330 (*H. nigricans* Belize/Panama, Table 1). Microsatellite data provided *F*_st_ estimates that ranged between 0.0011 (*H. unicolor* Honduras/Panama) and 0.0132 (*H. nigricans* Belize/Panama (Table 1). We note that sample sizes were relatively low for the RAD data, with a mean *n* of 17–25 per pairwise comparison (vs. 100–108 for microsatellites).

### Clustering analyses

**Figure 2:**
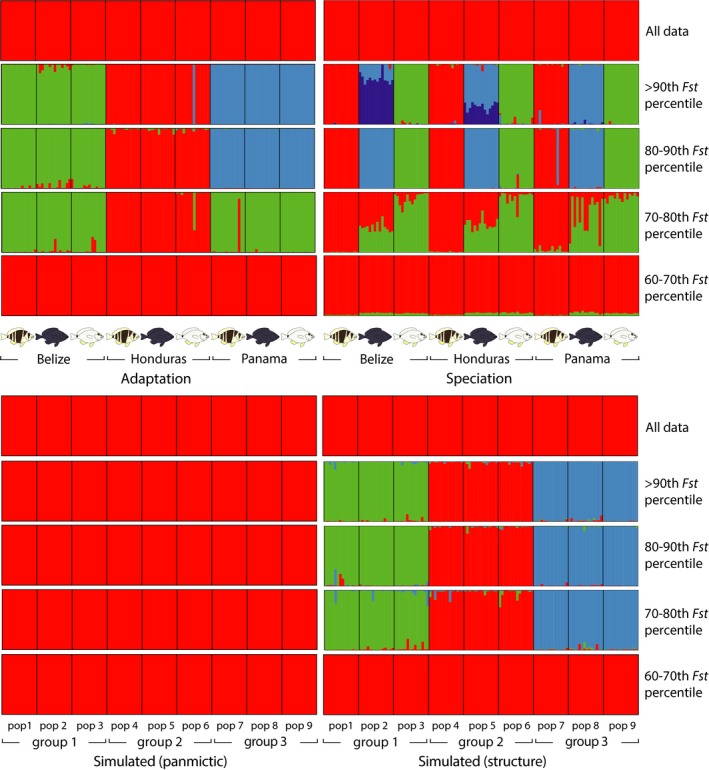
Clustering results for adaptation (among populations, Belize, Honduras, and Panama), speciation (among species, *Hypoplectrus puella*,* H. nigricans*, and *H. unicolor*, from Puebla et al. 2014), and simulated data (panmictic, migration rate *m *=* *0.5, and structure, migration rate *m *=* *0.02). In each case, the entire dataset (~40,000 SNPs) is presented above, followed by the SNPs above the 90^th^ *F*_st_ percentile, between the 80^th^ and 90^th^ *F*_st_ percentiles, between the 70^th^ and 80^th^ *F*_st_ percentiles, and between the 60^th^ and 70^th^ *F*_st_ percentiles (~8000 SNPs in each case). Details in Figure S1.

The clustering analyses are summarized in Figures 2 and S1. Using the entire dataset (41,690 SNPs), ln Pr(*X*|*K*) was systematically higher for *K *=* *1 than for any other value of *K* in the 10 replicate runs. Nevertheless, the black hamlets from Belize – the most differentiated sample according to the RAD and microsatellite *F*_st_ estimates – tended to form a distinct cluster in some runs with *K *=* *2. This pattern became consistent when removing loci with rare alleles (present in only one individual per location), in which case *K *=* *2 was identified as the best clustering solution (Fig. S2).

The SNP subsets from the 90^th^–100^th^, 80^th^–90^th^, and 70^th^–80^th^ *F*_st_ percentiles provided strong evidence of clustering. The highest mean ln Pr(*X*|*K*) corresponded to *K *=* *3 (90^th^–100^th^ and 80^th^–90^th^ percentiles) and *K *=* *2 (70^th^–80^th^ percentile). In each case, the Δ*K* statistic presented a clear peak at these *K* values, and the 10 replicate runs provided almost exactly identical groupings (including the three ‘misassigned' samples), although different seed numbers were used for each run. For the 90^th^–100^th^ and 80^th^–90^th^ percentiles, the three clusters corresponded to the three locations (Fig. 2). For the 70^th^–80^th^ percentile, the two clusters differentiated the Honduras samples from the Belize and Panama samples. No clustering was found with the SNPs from the 60^th^–70^th^ and 0–60^th^ percentiles. Similar patterns were obtained with more stringent filtering (loci present in ≥32 individuals per species instead of 15, data not shown).

### SNP trees

**Figure 3:**
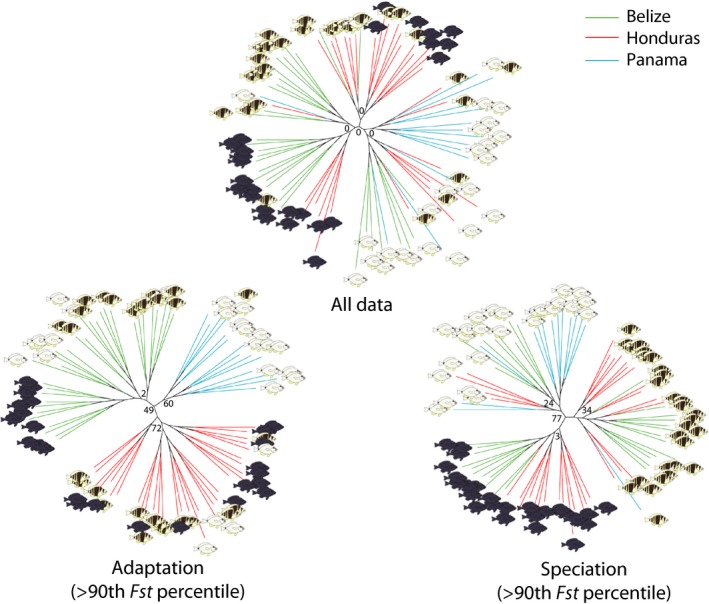
Maximum‐likelihood SNP trees for all data, adaptation (among populations, Belize, Honduras, and Panama, SNPs above the 90^th^ *F*_st_ percentile), and speciation (among species, *Hypoplectrus puella*,* H. nigricans*, and *H. unicolor*, SNPs above the 90^th^ *F*_st_ percentile, from Puebla et al. 2014). Bootstrap values within groups not shown.

A tendency to group samples by location and species was apparent when considering the entire dataset (Fig. 3), but the central node had a bootstrap support value of zero. The SNP subset from the 90^th^–100^th^ *F*_st_ percentile grouped samples by location with a bootstrap support value of 49. The SNP subsets from the 80^th^–90^th^, 70^th^–80^th^, and 60^th^–70^th^ percentile and below the 60^th^ percentile did not reveal any clear phylogenetic signal, with trees similar to these obtained with the entire dataset (data not shown).

### Linkage disequilibrium network analysis

**Figure 4:**
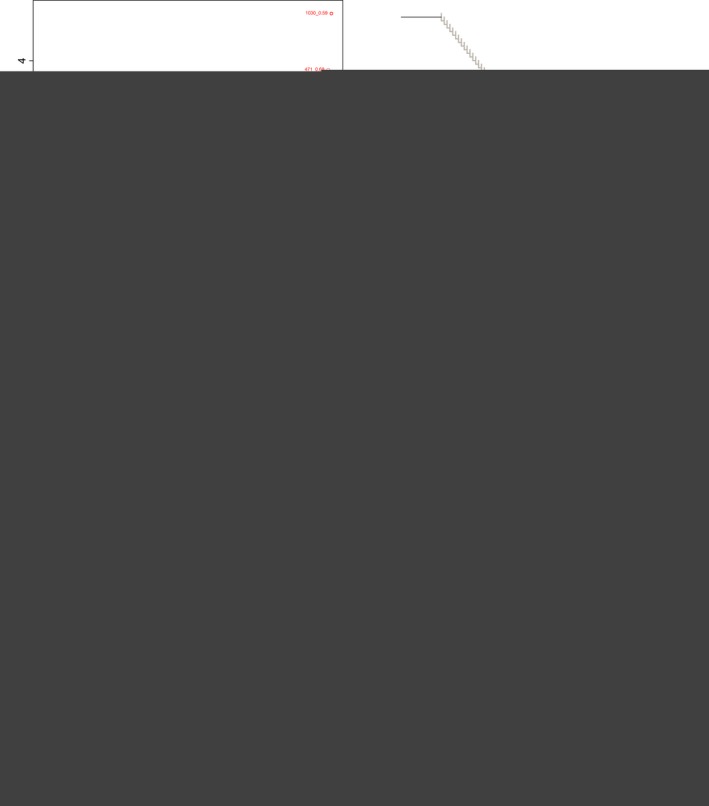
Results of the LDna analyses with *φ *= 4 and |*E*|min = 16. Five single‐outlier clusters (SOCs) were identified (in red). Two of them (1149 and 1030) did not appear to distinguish among the four samples, with global *F*_st_ estimates of 0.0027 and −0.0191, respectively. SOC 1030 consisted of tightly linked SNPs (median LD = 0.6 vs. ≤0.2 for the other four SOCs), which may reflect physical linkage (e.g., an inversion). The other three SOCs (471, 684, 923) presented higher *F*_st_ estimates among the four samples (0.0102, 0.0588, and 0.0253, respectively) and diffuse linkage (i.e., with a number of edges close to the number of loci). They tended to distinguish the black hamlets from Belize (the most differentiated sample) along the first DAPC axis and the barred hamlets from Honduras (471), barred hamlets from Belize (684), and black hamlets from Honduras (923) along the second axis, suggesting that these SOCs result from admixture LD.

A small proportion of SNPs (249 out of 10,734) presented LD values ≥ 0.8, the large majority of which involved a single pair of loci (Fig. S3). These may be on flanking regions of the same restriction site, as a single SNP per stack was used for these analyses. Larger clusters emerged, grew, and merged at lower LD values. Five single‐outlier clusters (SOCs) were identified with *φ* and |*E*|min set to four and 16, respectively (Fig. 4), and these same SOCs were also detected with various combinations of *φ* and |*E*|min (data not shown). The SOCs contained between nine and 43 loci each (total 127), representing 1.2% of the SNPs included in the analysis. Two of them (1149 and 1030) did not appear to distinguish the four samples, with global *F*_st_ estimates among samples of 0.0027 and −0.0191, respectively. SOC 1030 consisted of relatively tightly linked SNPs (median LD = 0.6 vs. ≤0.2 for the other four SOCs), which may reflect physical linkage (possibly an inversion). The other three SOCs (471, 684, 923) presented higher *F*_st_ estimates among the four samples (0.0102, 0.0588, and 0.0253, respectively) and diffuse linkage, that is, with a number of edges close to the number of loci. They tended to distinguish the black hamlets from Belize (the most differentiated sample) along the first DAPC axis and the barred hamlets from Honduras (471), barred hamlets from Belize (684), and black hamlets from Honduras (923) along the second axis, suggesting that these SOCs result from admixture LD.

### *F*_st_ outlier analyses

A total of 107 outliers were identified, with the prior odds for the neutral model set to 10, which represents 0.07% of the SNPs analyzed (Table 2). Three of these (38,220, 55,313, and 39,894) were identified in more than one species (repeated outliers) and all of them were ‘triple repeated outliers', that is, identified in *H. puella*,* H. nigricans*, and *H. unicolor* independently. Similar results (and the same repeated outliers) were obtained when running the *F*_st_ outlier analyses globally for each species instead of individually for each population pair (data not shown). Individual *F*_st_ estimates at the three outlier loci are highlighted in Figure 1A and detailed in Table 1. They were generally high, with global *F*_st_ estimates among populations within species ranging between 0.348 and one. The latter *F*_st_ estimate of one corresponded to a SNP on locus 38,220 that was fixed in Belize (C/C) versus Honduras and Panama (G/G) in the three species. A total of 19 outliers were identified with the prior odds for the neutral model set to 100, which represents 0.01% of the SNPs analyzed. Loci 38,220, 55,313, and 39,894 were identified as outliers here again, as well as in the other assemblies (Table S1). All loci were not included in all analyses as they were below the minimum coverage threshold in some populations (e.g., Table 1). Nevertheless, 78% of the outlier SNPs identified in one species were also considered in at least one other species, indicating that the small number of repeated outliers is not mainly due to a lack of coverage.

**Table 2:** Results of the *F*_st_ outlier analyses between Belize, Honduras, and Panama in *Hypoplectrus puella*,* H. nigricans*, and *H. unicolor*

Species	Location 1	Location 2	N. loci	N. (*n*) and ratio (%) of outliers			
				Prior odds=10		Prior odds=100	
				*n*	%	*n*	%
*H. puella*	Belize	Honduras	37,819	22	0.06	4	0.01
*H. nigricans*	Belize	Honduras	36,256	29	0.08	3	0.01
*H. unicolor*	Belize	Honduras	15,802	17	0.11	5	0.03
*H. puella*	Honduras	Panama	10,453	7	0.07	1	0.01
*H. nigricans*	Honduras	Panama	2145	1	0.05	0	<0.05
*H. unicolor*	Honduras	Panama	16,492	6	0.04	2	0.01
*H. puella*	Belize	Panama	10,293	6	0.06	0	<0.01
*H. nigricans*	Belize	Panama	2073	0	<0.05	0	<0.05
*H. unicolor*	Belize	Panama	27,847	19	0.07	4	0.01
		Total	159,180	107	0.07	19	0.01

A mini‐contig of 467 bp and mean coverage of 1033x was obtained for the repeated outlier locus 39,894. The consensus sequence mapped uniquely to an intron in the *Tpm4* gene in five teleosts, with E‐values ranging between 5E‐25 and 2E‐05 (Table 3). Similar blast searches for the other two repeated outlier loci (38,220 and 55,313) did not return strong hits. Blast hits of the nonrepeated outliers are presented in Table S3. Interestingly, one nonrepeated outlier (28,418) mapped to the same *Tpm4* locus identified above in several teleosts. The strongest hit was to the stickleback genome (2E‐31), to an intron situated 204 bp from exon 8 and 4826 bp from the repeated outlier.

**Table 3:** Results of the blast searches for the consensus sequence of the mini‐contig containing the repeated outlier SNP 39,894

Species	Alignment length (bp)	Identity (%)	E‐value	Annotation
Three‐spined stickleback (*Gasterosteus aculeatus*)	469	62	5E‐25	*tpm4* (intron, 1217 bp from exon 3)
Nile tilapia (*Oreochromis niloticus*)	361	65	5E‐23	*tpm4* (intron, 1320 bp from exon 3)
Southern platyfish (*Xiphophorus maculatus*)	134	75	8E‐16	*tpm4* (intron, 1502 bp from exon 3)
Spotted green pufferfish (*Tetraodon nigroviridis*)	103	78	2E‐11	*tpm4* (intron, 724 bp from exon 3)
Japanes pufferfish (*Takifugu rubripes*)	75	76	2E‐05	*tpm4* (intron, 1217 bp from exon 3)

### Randomizations and simulations

Two randomized datasets are illustrated in Figure 1C, D. Global *F*_st_ were estimated to 0.0001 and 0.0003, respectively, as opposed to 0.0042 for the real dataset. The distributions of SNP *F*_st_ estimates were similar to the real dataset (Fig. 1A), but slightly narrower and with a shorter tail. With the prior odds for the neutral model set to 10, a total of seven and 21 outliers (0.003 and 0.005% of the SNPs analyzed) were identified for each randomization, respectively, and no repeated outliers were found. For the ‘panmictic' scenario (*m *=* *0.5), the simulations provided global *F*_st_ estimates ranging between zero and 0.0005 (mean = 0.0002) and no *F*_st_ outliers. For the ‘structure' scenario (*m *=* *0.02), the simulations provided global *F*_st_ estimates ranging between 0.0031 and 0.0037 (mean = 0.0035) and 67 outliers, representing 0.02% of all the loci considered, and no repeated outliers. An example of each scenario is illustrated in Figure 1E, F. Results of the clustering analyses on the simulated datasets are illustrated in Figures 2 and detailed in S1. No clustering was observed in the ‘panmictic' scenario, even when considering the most differentiated SNPs, but clustering patterns similar to these observed in the real data were provided by the ‘structure' scenario (Figs. 2 and S1).

## Discussion

By specifically targeting the lower end of the ‘speciation continuum' (Seehausen et al. 2014), our sampling design provided the opportunity to not only explore the population genomic patterns of local adaptation (among allopatric populations) in three hamlet species but also contrast them to the population genomic patterns of speciation (among sympatric species, Puebla et al. 2014). The data revealed very similar levels of genomic divergence (*F*_st_ estimate = 0.0038–0.0042), *F*_st_ distributions (Fig. 1), proportions of *F*_st_ outliers (0.05–0.07%), and numbers of repeated outliers (1–3) for the two processes. In both cases, about 20% and 10% of the most differentiated SNPs distinguished populations and species consistently when considered together in the clustering and phylogenetic analyses, respectively (Figs. 2, 3). These results parallel the population genetic patterns reported in other recently diverged taxa such as East African cichlids (Seehausen et al. 2008; Wagner et al. 2012), Darwin's finches (De Leon et al. 2010), stick insects (Nosil et al. 2012), and the rough periwinkle (Ravinet et al. 2015), where divergence among populations within species or ecotypes can be comparable to divergence among species or ecotypes. Nevertheless, no other study that explicitly contrasts the population genomic patterns along these two axes of divergence comes to mind.

In hamlets, of the 32,681 most diverged SNPs (above the 80^th^ *F*_st_ percentile), only 7% were shared between populations and species comparisons. This pattern was equally true of outlier loci where, again, only 7% of the *F*_st_ outliers were shared between populations and species comparisons. In the same line, the three repeated outliers identified among populations differed from the single repeated outlier previously identified among species (Puebla et al. 2014). Different sets of loci appear therefore to be involved in local adaptation and speciation in *Hypoplectrus*, suggesting that genomes are diverging largely independently between allopatric populations versus sympatric species. This may be expected, given the nature of the two processes. Sympatric hamlet species are clearly differentiated in terms of color pattern, but are otherwise morphologically and ecologically extremely similar. Color pattern has been identified as an important trait for mate choice (Domeier 1994; Puebla et al. 2007, 2012a) and aggressive mimicry (Randall and Randall 1960; Thresher 1978; Puebla et al. 2007) in the group, and sympatric species are reproductively isolated from a behavioral perspective by strong assortative mating (Fischer 1980; Barreto and McCartney 2007; Puebla et al. 2007, 2012a). Nevertheless, gene flow is possibly ongoing through the rare hybrid spawnings observed in the field (<2% based on extensive observations), as no intrinsic incompatibilities have been observed in hybrid larvae (Whiteman and Gage 2007a).

Within species, allopatric populations present more subtle differences in morphology, diet, and behavior (Thresher 1978; Aguilar‐Perera 2004; Whiteman et al. 2007b; Holt et al. 2008; Puebla et al. 2008), with gene flow occurring through larval dispersal. Fertilization is external in the hamlets and both eggs and larvae are planktonic, with a pelagic larval duration that varies between 2 and 3 weeks (Domeier 1994; B. Victor, pers. comm.), allowing for substantial gene flow among distant locations.

Consistent with this expectation, we observed shallow levels of genetic structure in the hamlets, with a global *F*_st_ estimate of 0.0042 (0.0063 in *H. unicolor*, 0.0065 in *H. puella*, and 0.0131 in *H. nigricans*) among populations separated by >500 kilometers. Slightly lower *F*_st_ estimates are provided by microsatellites for the same species and populations (0.0034 global, 0.0032 in *H. puella*, and 0.0084 in *H. nigricans*), which is consistent with the higher diversity and larger sample size of the microsatellite dataset. The results are also consistent with the shallow Caribbean‐wide genetic structure reported for *H. puella* using microsatellites (*F*_st_ estimate = 0.0049, Puebla et al. 2009). Such low levels of population structure are common in marine species and are not surprising, given the life history of the hamlets. Considering patterns of genetic isolation by distance in *H. puella* and *H. nigricans*, we previously estimated a mean dispersal distance of 2–20 km for *Hypoplectrus* (Puebla et al. 2009, 2012b). Moreover, with an average census density of one adult per 150 m^2^ of reef in the three species and populations sampled in this study (O. Puebla, unpubl. data) and a simultaneous hermaphroditic mating system that implies a demographic sex ratio of 1:1 (Fischer 1981), the hamlets may have relatively large effective population sizes, which would contribute to maintain low levels of genetic structure. In agreement with the shallow genetic structure reported here, low levels of admixture linkage disequilibrium were observed, with <0.7% of SNPs involved in small and diffuse linkage clusters (Fig. 4).

### Local adaptation

The distribution of individual SNP *F*_st_ estimates indicates that a large fraction of the genome is undifferentiated among populations, with 64% of estimates <0.001 and a sharp mode close to zero (Fig. 1A). Accordingly, it is not surprising to observe no clear structure in the clustering and phylogenetic analyses when considering the entire dataset. Nonetheless, a tendency to group samples by populations and species is apparent in the phylogenetic analyses (Fig. 3A), and the most differentiated sample (the black hamlets from Belize) can be distinguished in the clustering analyses when removing rare alleles (Fig. S2). Thus, part of the genome appears to be differentiated among populations and species. This is further suggested by the long tail of the *F*_st_ distribution, which goes up to a value of one (Fig. 1A), versus 0.120 and 0.394 in the simulated (panmictic) and randomized datasets, respectively (Fig. 1C, E).

When considered together, the 20% and 10% most differentiated SNPs distinguish the three populations consistently for all species in the clustering and phylogenetic analyses, respectively (Figs. 2, 3). Simulations suggest that such a signal is not expected in the absence of genetic structure (Fig. 2), but we advise caution when interpreting patterns provided by the most diverged SNPs, as there is some circularity in the process of selecting these SNPs to then explore genetic structure, and some signal may result from this procedure with real data, even in the absence of genetic structure (e.g., Fig. S1, randomized dataset). This approach is therefore best suited to explore existing population genetic structure rather than to infer whether or not there is structure. In our case, it is clear from the microsatellite and RAD dataset that there are small differences among populations and species (Table 1). In this context, the most differentiated SNPs were selected to infer roughly what proportion of SNPs were consistently differentiated among populations, and compare them with the proportion and identity of SNPs that were consistently differentiated among species. Another situation in which this approach may be useful is to assign samples to populations when genetic structure is low (e.g., Benestan et al. 2015).

The occurrence of *F*_st_ outliers provides another line of evidence that part of the genome is differentiated. A total of 107 outliers were identified, representing 0.07% of the SNPs analyzed. Among these, three were identified as repeated outliers in *H. puella*,* H. nigricans*, and *H. unicolor* independently. In contrast, ≤21 outliers and no repeated outliers were found in the randomized and simulated (panmictic) datasets. Two of the three repeated outliers did not map to any known sequence, which illustrates the limitations of RAD sequencing as a tool to identify candidate genes in the absence of a reference genome. On the other hand, one repeated outlier mapped uniquely to an intronic region of the *Tpm4* gene in five teleosts (Table 3). In addition, another nonrepeated outlier also mapped to *Tpm4*, about 5000 bp from the repeated outlier in the stickleback genome. The identification of *Tpm4* as an *F*_st_ outlier in three hamlet species and at two loci independently suggests that it may be under selection and that it may play a role in local adaptation.

### Tpm4 as a candidate gene for local adaptation?

*Tpm4* codes for tropomyosin*,* a ubiquitous two‐stranded *α*‐helical coiled coil protein that is best known for its role in muscle contraction, but that is also present in nonmuscle cells in association with actin filaments (Perry 2001). Tropomyosin genes are highly conserved among vertebrates and six of them, including two *Tpm4* genes, have been identified in the Japanese pufferfish (*Takifugu rubripes,* Toramoto et al. 2004). Our repeated outlier (as well as the nonrepeated outlier) mapped exclusively to one of them in all the teleost genomes surveyed, suggesting that the assembly did not merge paralogs for this RAD locus.

*Tpm4* has been shown to be associated with diet‐induced plasticity in the pharyngeal jaw apparatus of the East African cichlid *Astatoreochromis alluaudi* (Gunter et al. 2013). It is tempting to speculate that the high levels of divergence found in *Tpm4* may be associated with local adaptation to different prey types in Belize, Honduras, and Panama. The hamlets are predators, with a diet that includes small shrimps, crabs, fishes, mysids, stomatopods, isopods, and polychaetes (Randall 1967), and a stomach content analysis including populations from Belize and Honduras evidenced significant differences in prey composition between populations (Whiteman et al. 2007b). Nevertheless, it is unclear to what extent these shifts translate into prey hardness differences that may drive similar effects to what is observed in East African cichlids. Temperature constitutes another, maybe more likely, potential selective factor that may act on tropomyosin through its effect on muscle function. This is particularly relevant for ectotherms, and *Tpm4* has been experimentally shown to be upregulated in skeletal muscle of the common carp (*Cyprinus carpio*) when exposed to cold temperatures (Gracey et al. 2004). Cold‐water fronts associated with the southerly extension of the North American high‐pressure system have been shown to occur yearly between December and February at the specific location where our Belize samples were collected (Koltes and Opishinki 2009). In this context, it is interesting to note that the *Tpm4* outliers (as well as the unidentified outlier with a *F*_st_ of one) were identified in pairwise comparisons involving Belize specifically (Belize‐Honduras and Belize‐Panama). We hypothesize that the outlier signal observed at the *Tpm4* locus is linked to local adaptation to periodic episodes of low temperatures in Belize. Fine mapping of the association between *Tpm4* and population differences is needed to refine this hypothesis and establish to what extent the high levels of genetic differentiation observed in *Tmp4* are due to reduced gene flow (Wu 2001) or low diversity (Cruickshank and Hahn 2014) in this region of the genome.

### False positives or parallel adaptation?

Among the 107 *F*_st_ outliers identified, three were found repeatedly in the three species, suggesting that they might be under selection and possibly involved in local adaptation. Sequencing coverage at these three loci (41x, 49x, and 88x) was substantially higher than the mean coverage of 31x, suggesting that high divergence does not result from allelic dropout (Gautier et al. 2013). Nevertheless, the significance of the remaining 104 outliers is more open to interpretation. On one hand, nonrepeated outliers may be false positives, a well‐known issue in genome scans (Pérez‐Figueroa et al. 2010; Vilas et al. 2012; Lotterhos and Whitlock 2014). Our RAD data, assembled de novo and filtered with moderate stringency, surely contain genotyping errors, null alleles, and under‐ or overmerged loci, all of which are expected to bias downstream analyses (Arnold et al. 2013; Davey et al. 2013; Gautier et al. 2013). Only 19 outliers were detected when applying more stringent parameters in the *F*_st_ outlier test, and 0.02% of the SNPs analyzed were identified as outliers in the simulated data with structure but no selection (vs. 0.07% in our dataset). This suggests that part of the nonrepeated outliers, possibly as many as 30% of them, may be false positives.

On the other hand, there are reasons to believe that at least a fraction of the nonrepeated outliers are real. First of all, the fact that our global *F*_st_ estimates are consistent with microsatellite data from the same species and populations and the relatively low levels of heterozygosity of the hamlets suggests that our *F*_st_ estimates are not disproportionately inflated by the occurrence of null alleles. In addition, the shallow levels of genomic structure reported here provide a very favorable scenario for the detection of loci under divergent selection (Pérez‐Figueroa et al. 2010). Finally, it is worth noting that filtering also introduces biases in the data (Arnold et al. 2013; Gautier et al. 2013; Huang and Knowles 2014; Mastretta‐Yanes et al. 2015), rendering the solution potentially as problematic as the problem itself. In sum, it is likely that part of the nonrepeated outliers might be real, and that parallel adaptation is occurring in the hamlets. The high proportion of nonrepeated outliers identified among populations reflects the patterns observed among hamlet species (Puebla et al. 2014) and *Littorina* ecotypes (Ravinet et al. 2015), suggesting that parallel evolution may be common in the sea.

### Concluding remarks

It is important to keep in mind that hamlets can be more diverged than the populations and species considered in this study, and that the distinction between local adaptation and speciation becomes blurred as populations and species diverge. For example, the hamlets from the Gulf of Mexico appear to be well diverged from similarly patterned Caribbean hamlets, and have been recently described as distinct species (Victor 2012; Tavera and Acero 2013). In this case, local adaptation to the specific conditions of the Gulf of Mexico and Caribbean may have contributed more to species divergence than color pattern. Within the Caribbean, some species such as the Maya hamlet (*Hypoplectrus maya*) or the masked hamlet (*Hypoplectrus providencianus*) present both distinct color patterns and high levels of endemism, suggesting that local adaptation and color pattern may have both played a role in species divergence. Ultimately, whether divergence is considered within the framework of local adaptation or speciation may reflect more a question of perspective and levels of divergence than a fundamental difference between the two processes. We conclude that marine populations may be locally adapted notwithstanding very shallow levels of genomic divergence, and that from a population genomic perspective, this process does not differ fundamentally from the early stages of speciation.

## Data Accessibility

RAD demultiplexed sequence data, SNP genotype calls and mini‐contig sequences: Dryad DOI: doi: 10.5061/dryad.nt722.

## Conflict of Interest

None declared.

## Supporting information


**Figure S1.** Clustering results for adaptation (between Belize, Honduras and Panama), simulated data (panmictic, migration rate *m *= 0.5 and structure, migration rate *m *= 0.02), and randomized data.
**Figure S2.** Clustering pattern obtained with *K *= 2 when considering all the data and removing rare alleles (present in only one individual per location).
**Figure S3.** Patterns of linkage disequilibrium among the 10,734 SNPs considered in the LD analysis.

**Table S1**. Summary of the five assemblies with different combinations of *m* (stack depth) and *M* (mismatch) parameters.

**Table S2.** Mean number of individuals sampled per site, observed and expected heterozygosity, nucleotide diversity (*π*) and *F*_is_ in the three locations considered in this study.

**Table S3.** Highest blast hits for the stacks containing non‐repeated outlier SNPs.
