# Supplementary material for: Population genomics of local adaptation versus speciation in coral reef fishes (Hypoplectrus spp, Serranidae)
Source: Ecol Evol. 2016 Feb 26;6(7):2109–24. doi: 10.1002/ece3.2028 (PMC4831444; doi:10.1002/ece3.2028)
Supplement: Supplementary file 1 — Figure S1. Clustering results for adaptation (between Belize, Honduras and Panama), simulated data (panmictic, migration rate m = 0.5 and structure, migration rate m = 0.02), and randomized data. Figure S2. Clustering pattern obtained with K = 2 when considering all the data and removing rare alleles (present in only one individual per location). Figure S3. Patterns of linkage disequilibrium among the 10,734 SNPs considered in the LD analysis. [file ECE3-6-2109-s001.doc]

**Population genomics of local adaptation versus speciation in coral reef fishes (*Hypoplectrus* spp, Serranidae)**

**Supporting Information**

Sophie Picq, W. Owen McMillan, Oscar Puebla

**Supporting Table S1**. Summary of the 5 assemblies with different combinations of *m* (stack depth) and *M* (mismatch) parameters. As expected (Catchen *et al*. 2013), the number of stacks decreases with increasing *m* and *M* parameter values. Assuming a 1Gb genome typical of many serranids and a GC content of 41% (from the paired-end reads), one would naively expect 6,459 *Sbf*I cut sites in *Hypoplectrus* $\left(0.41/2\right)^{6}\times\left(0.59/2\right)^{2}\times10^{9}$. This is four times less than the 26,906 sites suggested by our main assembly (*m*=3 *M*=2). Such discrepancies are not rare (e.g. 3,221 expected *Sbf*I cut sites versus 22,830 observed in the stickleback genome, Hohenlohe *et al*. 2010), and stress the importance of performing a pilot study when planning RAD studies on an organism for the first time or with a new restriction enzyme. References in the main text.

**Supporting Table S2.** Mean number of individuals sampled per site, observed and expected heterozygosity, nucleotide diversity (*π*) and *F_is_* in the three locations considered in this study.

**Supporting Table S3.** Highest blast hits for the stacks containing non-repeated outlier SNPs. Only hits with a E-value <1E-06 are shown.

**
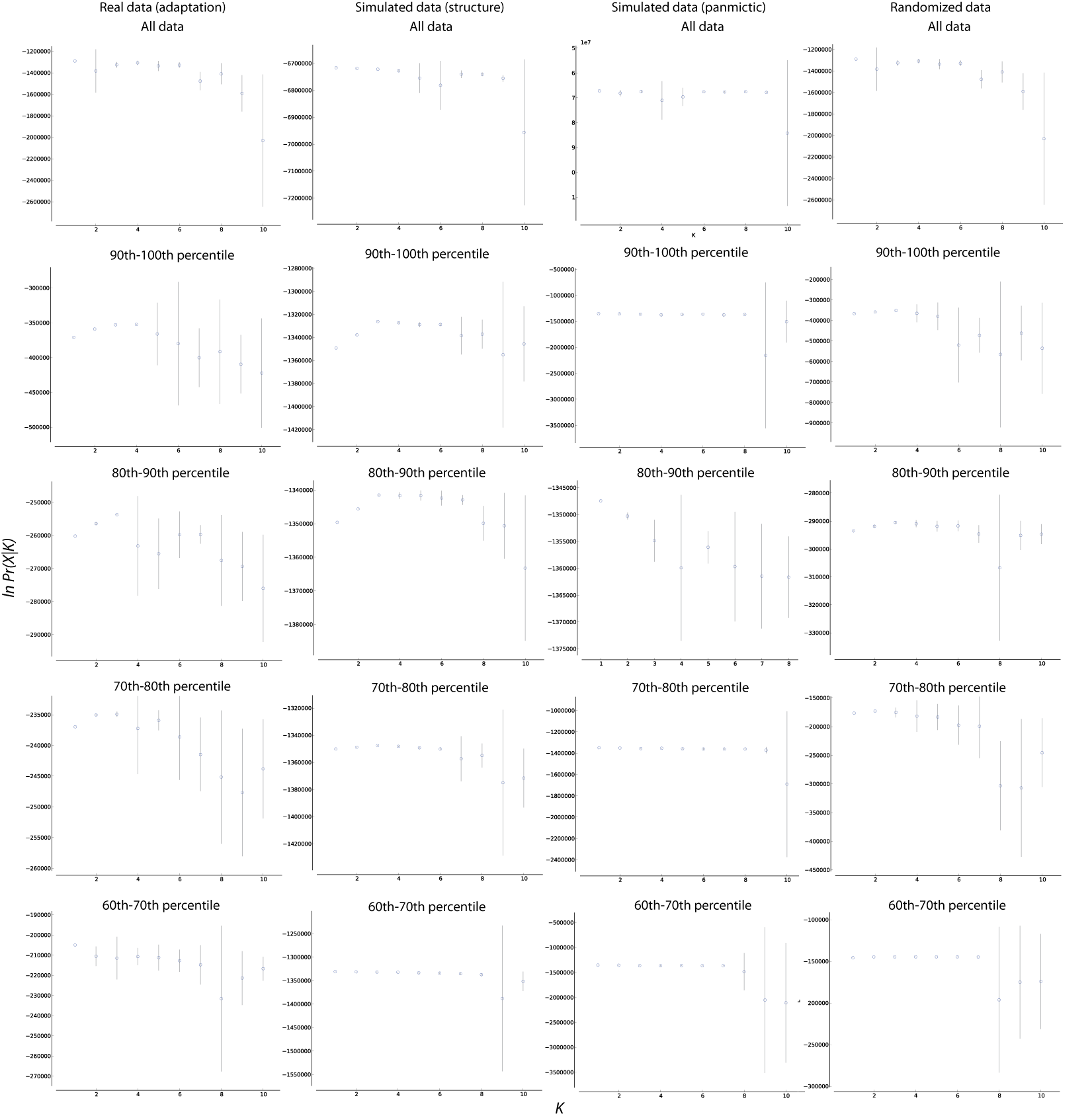
**

**Supporting Figure S1.** Clustering results for adaptation (between Belize, Honduras and Panama), simulated data (panmictic, migration rate *m*=0.5 and structure, migration rate *m*=0.02), and randomized data. In each case the entire dataset (~40,000 SNPs) is presented above, followed by the SNPs above the 90^th^ *F_st_* percentile, between the 80^th^ and 90^th^ *F_st_* percentiles, between the 70^th^ and 80^th^ *F_st_* percentiles, and between the 60^th^ and 70^th^ *F_st_* percentiles (~8,000 SNPs in each case). No clustering was found when considering the SNPs below the 60^th^ percentile (data not shown).

**
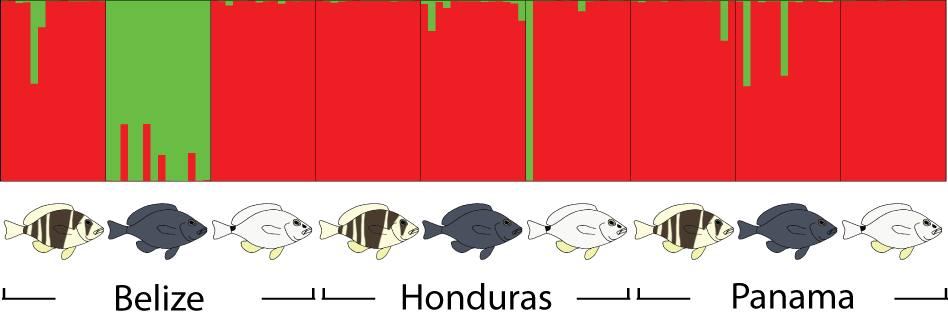
**

**Supporting Figure S2.** Clustering pattern obtained with *K*=2 when considering all the data and removing rare alleles (present in only one individual per location). The same pattern was observed in the 10 replicate runs, although each run was started with a different seed number.


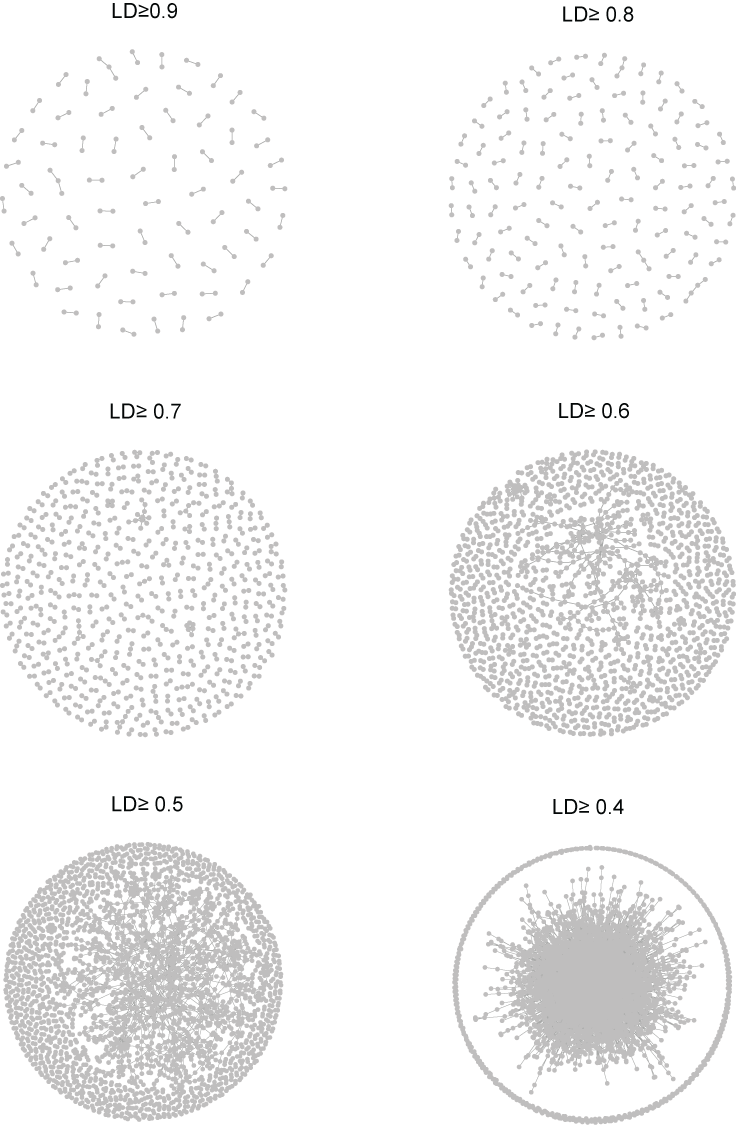


**Supporting Figure S3.** Patterns of linkage disequilibrium among the 10,734 SNPs considered in the LD analysis. Vertices represent loci and edges LD values that are above a given threshold (indicated above each plot). A small proportion of SNPs (249 out of 10,734) present LD values ≥ 08, the large majority of which involve a single pair of loci. These may be on flanking regions of the same restriction sites, as a single SNP per stack was used for these analyses. Larger clusters emerge, grow and merge at lower LD values.
